# Accelerated vaccine rollout is imperative to mitigate highly transmissible COVID-19 variants

**DOI:** 10.1016/j.eclinm.2021.100865

**Published:** 2021-04-25

**Authors:** Pratha Sah, Thomas N. Vilches, Seyed M. Moghadas, Meagan C. Fitzpatrick, Burton H. Singer, Peter J. Hotez, Alison P. Galvani

**Affiliations:** a Center for Infectious Disease Modeling and Analysis (CIDMA), Yale School of Public Health, New Haven, CT, USA; b Agent-Based Modelling Laboratory, York University, Toronto, Ontario M3J 1P3 Canada; c Center for Vaccine Development and Global Health, University of Maryland School of Medicine, 685 W Baltimore St, Baltimore, MD 21201, USA; d Emerging Pathogens Institute, University of Florida, Gainesville, FL 32610, USA; e National School of Tropical Medicine, Baylor College of Medicine, Houston, TX, USA

## Abstract

**Background:**

More contagious variants of SARS-CoV-2 have emerged around the world, sparking concerns about impending surge in cases and severe outcomes. Despite the development of effective vaccines, rollout has been slow. We evaluated the impact of accelerated vaccine distribution on curbing the disease burden of novel SARS-CoV-2 variants.

**Methods:**

We used an agent-based model of SARS-CoV-2 transmission and vaccination to simulate the spread of novel variants with S-Gene Target Failure (SGTF) in addition to the original strain. We incorporated age-specific risk and contact patterns and implemented a two-dose vaccination campaign in accord with CDC-recommended prioritization. As a base case, we projected hospitalizations and deaths at a daily vaccination rate of 1 million doses in the United States (US) and compared with accelerated campaigns in which daily doses were expanded to 1.5, 2, 2.5, or 3 million.

**Findings:**

We found that at a vaccination rate of 1 million doses per day, an emergent SGTF variant that is 20–70% more transmissible than the original variant would become dominant within 2 to 9 weeks, accounting for as much as 99% of cases at the outbreak peak. Our results show that accelerating vaccine delivery would substantially reduce severe health outcomes. For a SGTF with 30% higher transmissibility, increasing vaccine doses from 1 to 3 million per day would avert 152,048 (95% CrI: 134,772–168,696) hospitalizations and 48,448 (95% CrI: 42,042–54,285) deaths over 300 days. Accelerated vaccination would also prevent additional COVID-19 waves that would otherwise be fuelled by waning adherence to non-pharmaceutical interventions (NPIs).

**Interpretation:**

We found that the current pace of vaccine rollout is insufficient to prevent the exacerbation of the pandemic that will be attributable to the novel, more contagious SARS-CoV-2 variants. Accelerating the vaccination rate should be a public health priority for averting the expected surge in COVID-19 hospitalizations and deaths that would be associated with widespread dissemination of the SGTF variants. Our results underscore the need to bolster the production and distribution of COVID-19 vaccines, to rapidly expand vaccination priority groups and distribution sites.

**Research in context**

**Evidence before this study**

At least three new SARS-CoV-2 variants with S-Gene Target Failure (SGTF) have been detected in the US. Multiple lines of evidence indicate that these variants spread more rapidly than the original strain. Although fewer than 7000 cases caused by these variants have been reported in the US as of March, 21 2021, their incidence is predicted to double every 10 days. Vaccines are the best defense against COVID-19, however, the rate of vaccination has been slower than expected in the US. The current pace of vaccination might be inadequate to mitigate a surge in cases as the new variants become widespread. We searched PubMed from inception through March 21, 2021, for articles using the search terms “SARS-CoV-2”, “novel coronavirus”, “coronavirus 2019”, “COVID-19”, “COVID 2019” AND “variants”, “variant”, “SGTF”, “S-Gene Target Failure” AND “vaccine”, “vaccination” AND “United States”, “US”. Our search yielded one relevant published article which investigated the impact of current pace of vaccination on the emergence of the B.1.1.7 variant in the United States. We found no published articles assessing the impact of accelerated vaccination in the United States to curb the spread of new SARS-CoV-2 variants.

**Added value of this study**

Our results show that the dominance of new variants with higher transmissibility is inevitable within a few weeks of introduction, exacerbating the pandemic toll in the United States. The current pace of vaccination, while has increased steadily from the start of the vaccination campaigns, would still be insufficient to mitigate increased hospitalizations and fatalities as the new variants take over. We show that accelerating vaccine rollout would not only save lives, but also prevent another wave of COVID-19 if adherence to non-pharmaceutical interventions such as social distancing declines over time.

**Implications of all the available evidence**

The results of this study provide evidence to policymakers in the United States and globally that the production and distribution of COVID-19 vaccines must be bolstered to protect against the novel, more transmissible virus variants.

Alt-text: Unlabelled box

## 1 Introduction

As the coronavirus disease 2019 (COVID-19) pandemic continues to spread, disrupting healthcare and the economy around the world even a year after its emergence, new variants of the virus have emerged [1], [2], [3]. The B.1.1.7 variant of SARS-CoV-2, first reported in the United Kingdom in the fall of 2020, has quickly become the dominant circulating strain in that country and has already been detected in numerous countries including the United States [4]. Cases caused by other variants B.1.351 and P.1, first reported in South Africa and Brazil, respectively, have also been detected in the US [4].

These new variants exhibit a multiplicative transmissibility up to 74% higher than the original strain, [5], [6], [7] and higher case fatality rates [8,9]. While the B.1.1.7 variant accounted for only 3.6% of new cases in the US by the last week of January, 2021, it was predicted to become the dominant variant in many states before April 2021 [10,11]. Therefore, as we head into the spring months we might expect an upturn in the numbers of new COVID-19 cases in the US and a rise in deaths.

The new variants carry multiple mutations concentrated in the segment of the viral genome that codes for the spike protein, targeted by several vaccine platforms (e.g., Pfizer/BioNTech, Moderna, Johnson and Johnson, Novavax and Oxford-AstraZeneca) [12]. Initial evidence from clinical trials suggests that these vaccines may have reduced efficacy in preventing mild to moderate COVID-19 for infections caused by the B.1.351 variant [13]. As high levels of virus continue to circulate, emergence and spread of further mutations may erode vaccine efficacy, potentially compromising efforts to control the pandemic.

Vaccines have the ability to save lives and avert hospitalizations by suppressing viral circulation, curtailing transmission of existing variants and preventing the emergence of more transmissible ones [14]. However, rollout in the United States has been slower than expected. During the first month of vaccine availability, less than 5.5 million people received their first dose of vaccine, falling short of the goal to vaccinate 20 million people set by US officials [15]. Under the new administration, the vaccination rate has quickly ramped up from 1 million doses per day in January to the current average of 2 million vaccine doses administered per day as of March 2021. However, this pace may still be insufficient to reduce hospitalization and death as the new variants become widespread.

Here, we evaluated the impact of accelerating vaccination rollout to curb the spread of new SARS-CoV-2 variants. To do so, we extended our previous agent-based model of a COVID-19 outbreak [16] to consider transmission dynamics of both the original strain and new SARS-CoV-2 variants with S-gene target failure (SGTF). We compared scenarios with increased vaccination in the US to 1.5, 2, 2.5, and 3 million doses per day from baseline 1 million doses a day. We find that widespread transmission of the SGTF variants in the US would significantly increase COVID-19 cases, hospitalizations and deaths. Accelerating the US COVID-19 vaccination ahead of these variants would avert a catastrophic toll in both hospitalizations and deaths.

## 2 Methods

### 2.1 Model structure

We extended our previous agent-based model of COVID-19 transmission and vaccination [16] to include new variants of SARS-CoV-2 with different transmissibilities in addition to the original strain. This modelling framework allowed us to implement disease course and vaccine efficacy timelines at the individual level in different age groups (Appendix, Tables A1, A3). The natural history of COVID-19 was implemented with epidemiological classes for susceptible; latently infected (not yet infectious); asymptomatic (and infectious); pre-symptomatic (and infectious); symptomatic (and infectious) with either mild or severe illness; recovered; and dead. To incorporate age-specific risk factors, contact patterns, and vaccine allocations, we stratified the model population into six age groups of 0–4, 5–19, 20–49, 50–64, 65–79, and 80+ years based on US census data (Appendix Table A1) [17], [18], [19]. Daily contacts between individuals were sampled from a negative-binomial distribution parameterized using an empirically-determined age-specific contact network (Appendix, Table A2) [20,21].

### 2.2 Disease dynamics

Transmission was probabilistically implemented as a result of contacts between susceptible and infected individuals. We parameterized the infectivity of asymptomatic, mild symptomatic, and severe symptomatic individuals to be 26%, 44%, and 89% relative to the pre-symptomatic stage [22], [23], [24]. We assumed that these relative infectivities remained the same for all SARS-CoV-2 variants. Regardless of viral variant, the incubation period was sampled from a Gamma distribution with a mean of 5.2 days [25]. An age-dependent proportion of infected individuals went through a pre-symptomatic stage with a mean duration of 2.3 days [23,26], and developed symptomatic disease with a mean duration of 3.2 days [27,28]. Those who did not develop symptomatic disease remained asymptomatic until recovery, with a mean infectious period of 5 days [27,28]. Durations of asymptomatic, pre-symptomatic, and symptomatic stages were sampled for each individual independently from their respective Gamma distributions. We assumed that recovery from infection conferred immunity against reinfection for at least one year. Disease-specific parameters are summarized in Table 1.

**Table 1:** Description of model parameters and their estimates.

Description		0–4	5–19	20–49	50–64	65–79	80+	Source

Transmission probability per contact during pre-symptomatic stage		0.0385						Calibrated to R=1.1 [50]
Incubation period (days)		LogNormal(shape: 1.434, scale: 0.661)						[25]
Asymptomatic period (days)		Gamma(shape: 5, scale: 1)						[27,28]
Pre-symptomatic period (days)		Gamma(shape: 1.058, scale: 2.174)						[23]
Infectious period from onset of symptoms (days)		Gamma(shape: 2.768, scale: 1.1563)						[23,27]
Proportion of infections that are asymptomatic		0.30	0.38	0.33	0.33	0.19	0.19	[51], [52], [53], [54]
Proportion of symptomatic cases that exhibit mild symptoms		0.95	0.90	0.85	0.60	0.20	0.20	[29,30]
Proportion of cases hospitalized with one or more comorbidities		37.6%						[31,32]
	Non-ICU	67%						
	ICU	33%						
Proportion of cases hospitalized without any comorbidities		9%						[31,32]
	Non-ICU	75%						
	ICU	25%						

### 2.3 Infection outcomes

We assumed that asymptomatic and mild symptomatic cases recover from infection without hospitalization. A proportion of those with severe disease were hospitalized within 2–5 days of symptom onset [29,30], and thereafter were removed from the chain of disease transmission. We also assumed that all symptomatic cases who were not hospitalized self-isolated within 24 h of symptom onset, and reduced their number of daily contacts by an average of 74%. This reduction of contacts was derived from a representative sample population during COVID-19 lockdown [20]. Intensive care unit (ICU) and non-ICU hospitalization rates were stratified by both age and comorbidities, parameterized by clinical and epidemiological data [18,31,32]. The risk of death as an outcome of severe disease was age-dependent, and the hazard of death associated with infection by SARS-CoV-2 variants with SGTF was 30% higher relative to those without SGTF [9].

### 2.4 Vaccination

We implemented a two-dose vaccination campaign with prioritization set sequentially to: (i) healthcare workers (5% of the total population) [33], adults with comorbidities, and those aged 65 and older; and (ii) other individuals aged 16–64 [34,35]. In our model, the minimum age-eligibility was 16y for Pfizer-BioNTech vaccines, and 18y for Moderna vaccines. We used an average of reported vaccine doses distributed since the start of vaccination to simulate the base-case scenario for the roll-out strategy. In the base-case, the daily number of vaccine doses administered was 8 per 10,000 population for the first 20 days of vaccination campaigns, starting on December 12, 2020. It then increased to 20 doses for the next 20 days and plateaued at 30 vaccine doses daily per 10,000 population, corresponding to ~1 million vaccine doses per day in the entire US population [15]. We compared the base-case scenario to vaccination campaigns in which the number of daily doses starting on day 80 would be increased from 1 million to 1.5, 2, 2.5, or 3 million doses per day. These rates correspond to 30, 45, 60, 75, and 90 vaccine doses per day per 10000 population.

Our base-case specified Moderna vaccines with an interval of 28 days between the first and second doses [36]. In an additional scenario analysis, we considered Pfizer-BioNTech vaccines with an interval of 21 days as tested in clinical trials [37]. We conducted a review of published studies from clinical trials, FDA briefing documents, and the nationwide studies for the effect of vaccine in prevention infection, symptomatic disease, and severe disease (Appendix, Table A3) to parameterize the model for vaccine efficacy of each dose at the individual [37], [38], [39], [40], [41], [42].

### 2.5 Transmissibility

To determine the transmission probability of the pre-existing SARS-CoV-2 variant without SGTF, we calibrated the model to an effective reproduction number of 1.1 [43], which accounts for NPIs at the start of simulations with vaccination. We then introduced a variant of SARS-CoV-2 with SGTF 40 days after the start of vaccination. We applied a multiplicative factor for the increased relative transmissibility (referred to as RT) of the SGTF variant in the range 10–70%, compared to the pre-existing variant [6].

### 2.6 Model implementation

We simulated the model with a population of 10,000 individuals across a time horizon of 300 days, assuming 10% pre-existing immunity at the start of vaccination [44,45]. To incorporate the age distribution of pre-existing immunity in the population, we simulated the model in the absence of vaccination and determined the infection rates in different age groups when the overall attack rate reached 10%. The distribution of this immunity was used to parameterize the initial population at the start of vaccination. We then ran the simulations with different scenarios of SGTF variant transmissibility and vaccination speed (i.e., daily number of vaccine doses), evaluating expected incidence, hospitalizations and deaths. Point estimates for results were generated by averaging 1000 independent Monte-Carlo realizations in each scenario, and credible intervals (CrI) were generated using the bias-corrected and accelerated bootstrap method (with 500 replications). Assuming constant risks across different geographic regions, a nonparametric approach was used to extrapolate the estimates for the US population. The model was implemented in Julia, which is an open-source, high-performance, dynamic programming language that allows rapid analysis of computationally intensive problems, such as agent-based modelling. The simulation codes are available at: https://github.com/thomasvilches/two_strains.

### 2.7 Role of the funding source

The funders of the study had no role in study design, data collection, data analysis, data interpretation, or writing of the report.

## 3 Results

**Fig. 1:**
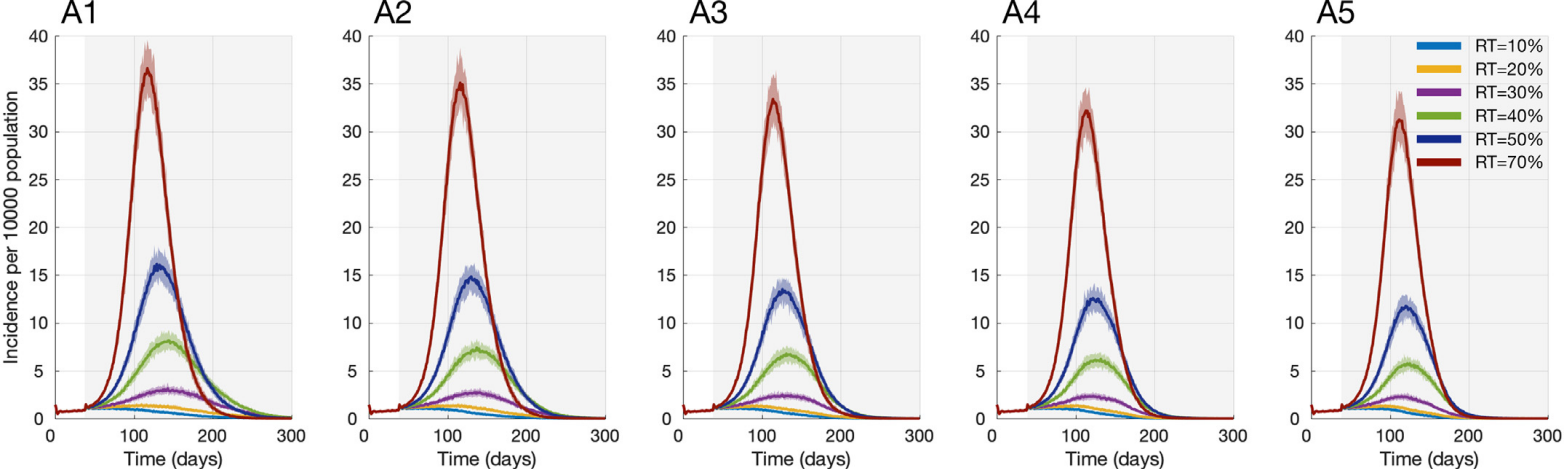
Projected incidence of infection per 10,000 population from the start of vaccination with different relative transmissibility of SGTF variants. Maximum daily Moderna vaccines administered are either (A1) 1 million; (A2) 1.5 million; (A3) 2 million; (A4) 2.5 million; or (A5) 3 million doses, per 10000 population. Vaccine efficacy was assumed to be the same against the original strain and variants with SGTF.

At a vaccination rate of 1 million doses per day, we found that emergence of a SARS-CoV-2 variant that is 10–70% more transmissible than the original variant would lead to an average of 1.24–36.6 cases per 10,000 population (Fig. 1), corresponding to 40,900–1,208,000 cases per day at the peak in the US. This transmissibility range represents an effective reproduction number (R_SGTF_) between 1.21 and 1.87 for the SGTF variant. With an R_SGTF_ above 1.3, the variant would become dominant in case incidence within 14–60 days after introduction. Dominance shifts earlier as the R_SGTF_ increases (Appendix, Fig. A1). For a 20% or more transmissible SGTF variant, 55–99% of cases at the outbreak peak would be caused by this variant (Appendix, Fig. A2).

**Fig. 2:**
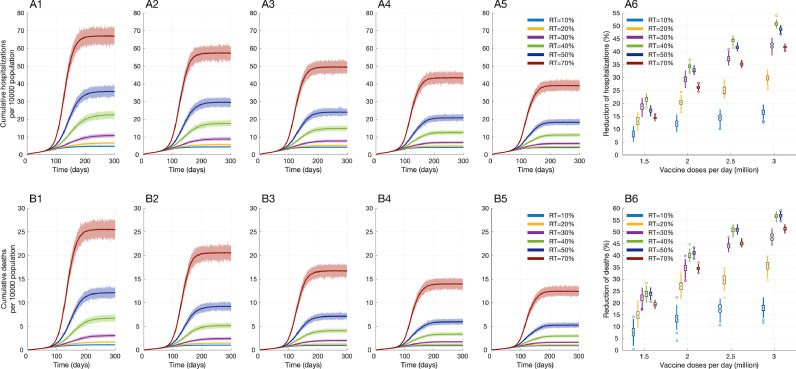
Projected cumulative hospitalizations (A1-A5) and deaths (B1-B5) per 10000 population for 300 days since the start of vaccination with different relative transmissibility of SGTF variants. Vaccine rollouts are with (A1,B1) 1 million; (A2,B2) 1.5 million; (A3,B3) 2 million; (A4,B4) 2.5 million; and (A5,B5) 3 million doses per day. Panels A6 and B6 represent the reduction of hospitalizations and deaths achieved by increasing the number of daily Moderna vaccine doses from 1 to 3 million doses. Vaccine efficacy was assumed to be the same against the original strain and variants with SGTF.

We found that increasing the rate of vaccine delivery would substantially reduce severe health outcomes (Appendix, Table A5). For example, with 1 million vaccine doses delivered per day and introduction of a SGTF variant that is 30% more transmissible, we project a total of 10.9 (95% CrI: 10.0–11.7) hospitalizations and 3.1 (95% CrI: 2.8–3.3) deaths per 10,000 population over 300 days (Fig. 2). These estimates correspond to 359,204 (95% CrI: 330,924–385,539) hospitalizations and 100,960 (95% CrI: 91,674–109,494) deaths across the US. Doubling the vaccination rate to 2 million doses per day would reduce total hospitalizations to 7.7 (95% CrI: 7.1–8.2) and deaths to 2.0 (95% CrI: 1.8–2.2) per 10,000 population, corresponding to mean reductions of 29.4% and 35.5%, respectively. Expanding the vaccination rate to 3 million doses per day would further reduce total COVID-19 hospitalizations and deaths to 6.3 (95% CrI: 5.8–6.7) and 1.6 (95% CrI: 1.5–1.7) per 10,000 population, respectively, equivalent to mean reductions of 42.2% and 48.8%, when compared to the scenario of 1 million vaccine doses per day (Fig. 2). Across the US, we project that increasing the vaccine doses from 1 to 3 million per day under this scenario would avert 152,048 (95% CrI: 134,772–168,696) hospitalizations and 48,448 (95% CrI: 42,042–54,285) deaths over 300 days.

At the population level, the benefit of ramping up vaccination efforts would be greatest when relative transmissibility of SGTF variants is high (Fig. 2 and Appendix, Figure A4). Expanding vaccination from 2 to 3 million doses per day, for example, is projected to avert 7174 (95% CrI: 3927–11,022) and 1694 (95% CrI: 165–3234) hospitalization and deaths in the US population, respectively, if the SGTF variant was 10% more transmissible than the original strain. In contrast, a similar expansion of vaccination for a variant that is 50% more transmissible would avert 189,113 (95% CrI: 167,079–208,659) and 62,647 (95% CrI: 54,219–71,676) hospitalizations and deaths, respectively.

**Fig. 3:**
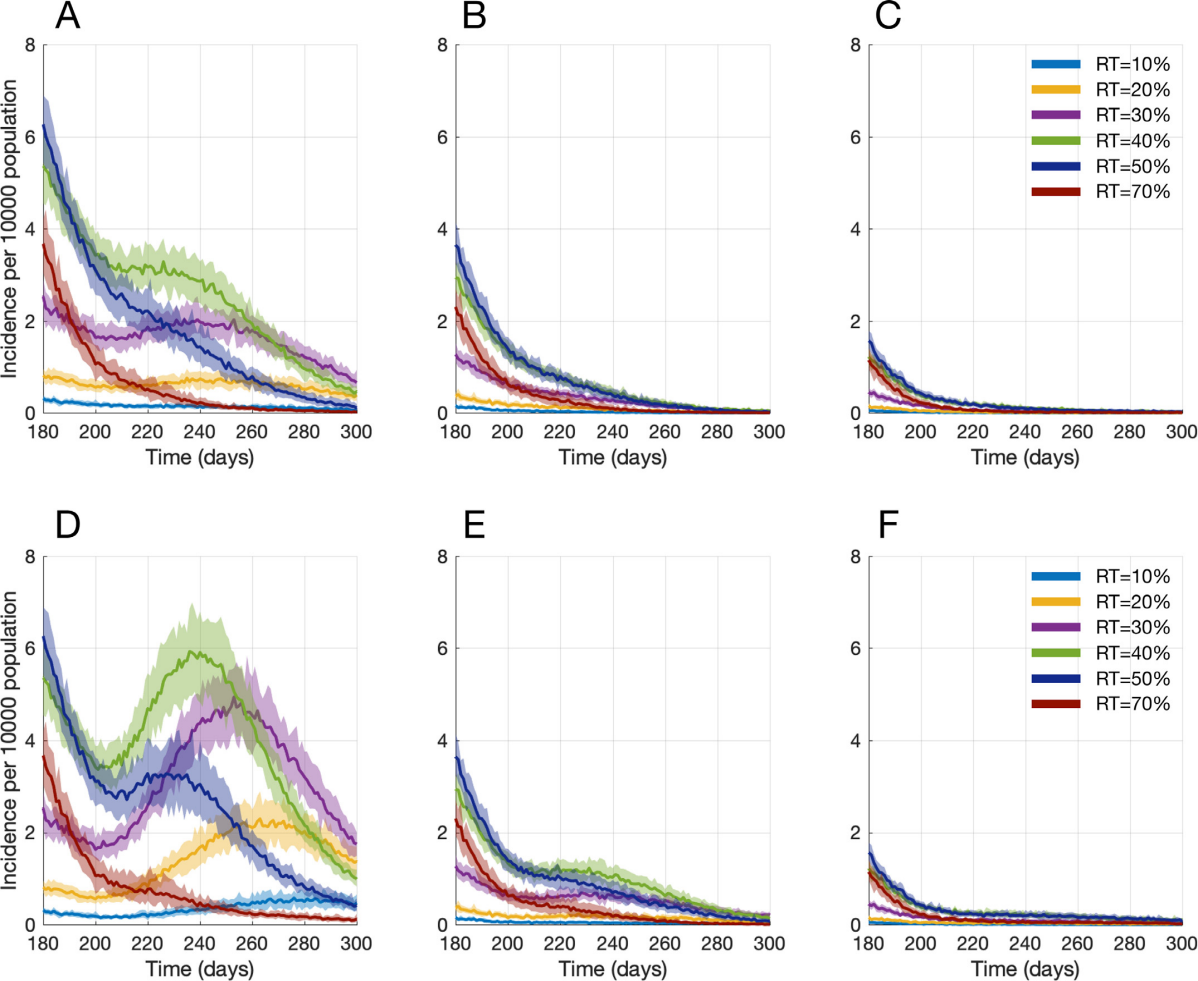
Projected incidence of infection per 10,000 population from the start of vaccination with different relative transmissibility of SGTF variants. The number of daily contacts increased by 20% (A,B,C), and 40% (D,E,F) 200 days after the start of vaccination. Maximum daily Moderna vaccines administered are either 1 million (A,D); 2 million (B,E); or 3 million doses (C,F) in the entire US population. Vaccine efficacy was assumed to be the same against the original strain and variants with SGTF.

Accelerated vaccination would also impact subsequent waves of COVID-19 when adherence to NPIs such as social distancing reduces over time. To demonstrate this effect, we allowed for a 20% or 40% increase in the number of daily contacts starting 200 days into the vaccination campaign, when cases in all scenarios are expected to be declining. Under the scenario of 1 million vaccine doses per day, such an increase in daily contacts could lead to an additional infection wave or prolong the outbreak duration by several months (Fig. 3). We found that improving the vaccination rate to 2 million doses per day mitigates the severe outcomes during subsequent waves. Accelerating the vaccination rate to 3 million doses per day would entirely offset the effect of decreased adherence to social distancing, and prevent additional COVID-19 waves even if daily contacts were increased by 40% (Fig. 3).

These results were obtained by assuming equal vaccine efficacy against SGTF variants and the original strain. We found that if vaccine efficacy against SGTF variants is reduced by 20% compared to the original strain, accelerating vaccine rollout becomes even more important in mitigating severe health outcomes and preventing additional COVID-19 waves that could arise from waning adherence to NPIs (Appendix Figures A5 and A8). For example, under the scenario of reduced vaccine efficacy against the new variants with 30% higher transmissibility, increasing the vaccination rate from 1 million to 3 million doses would avert 175,907 (95% CrI: 157,245–194,337) hospitalizations and 56,646 (95% CrI: 49,566–63,624) deaths. Compared to the scenario with equal vaccine efficacy against both the original and SGTF variants, this corresponds to a 15.7% and 16.9% greater reduction in hospitalizations and deaths, respectively.

## 4 Discussion

The emergence of highly transmissible SARS-CoV-2 variants will likely exacerbate outbreaks and undermine the control that could otherwise be gained by vaccination. Our results indicate that a SGTF variant would become dominant within a few weeks of being introduced, depending on its transmissibility relative to the current circulating strain. Our findings are consistent with the results of two previous studies projecting that the B.1.1.7 lineage will become the predominant variant in the US by March 2021 [10,11].

As the new variants inevitably become dominant, we show that the speed of vaccine deployment is critical to mitigating the projected increase in hospitalizations and deaths. With a SGTF variant that is 30% more transmissible than the original strain, for instance, we project that doubling the daily vaccination rate from 1 million doses to 2 million doses would avert 29.4% of hospitalizations and 35.5% of deaths. Tripling the vaccination rate to 3 million doses per day would cut both hospitalizations and death by nearly half compared with a daily vaccination rate of 1 million doses. We found that diminished vaccine efficacy against variants would necessitate an even more rapid increase in the vaccine coverage to offset the ensuing increase in disease burden.

Our study also highlights that rapid rollout has benefits beyond dampening the initial surge of infection and reducing severe health outcomes generated by novel variants. With no vaccines available during the initial outbreak of COVID, various NPIs such as social distancing, quarantine and usage of masks were adopted by countries worldwide. After almost a year of such measures, pandemic fatigue is eroding adherence to these measures [46,47]. Encouragingly, our study shows that accelerated vaccine rollout is capable of mitigating future waves that would result from this erosion, underscoring the urgency. Our results are also supported by a recent study demonstrating that accelerated vaccination rollout in England could avert a resurgence of cases following the relaxation of NPIs [48].

As with all models, our results should be considered within the context of study assumptions. First, we included only one variant of SARS-CoV-2 in our model in addition to the original strain. Several variants of the virus with SGTF have already been identified and may circulate concurrently with different transmissibilities. However, our projections cover a range of transmissibility which encompass the expected dynamics in scenarios with multiple variants. Second, our parameter values for transmissibility and risk of death for the SGTF variants are derived from early reports of the B.1.1.7 variant in the UK, but these estimates may vary across different populations or settings. Despite these considerations, our study unequivocally suggests that a substantial increase in vaccination rates would be needed to avert a devastating surge propelled by highly transmissible variants.

As countries around the world mobilize for COVID-19 vaccination, surveillance to detect the emergence of novel variants is critically important to quantify their transmissibility and potential effects on vaccine efficacy. In the event of antigenic escape, new generations of vaccines such as those using an mRNA platform can be updated much faster than traditional vaccines [49]. Efforts in this direction have already begun by Moderna, Pfizer and AstraZeneca to develop booster shots tailored to emerging variants. Concomitantly, the pace of rollout affects the probability that even more variants will emerge. Harmful mutations are more likely to arise when viral replication goes unchecked, and so swift global control not only provides benefits now but reduces future risk.

Our results demonstrate that the current pace of vaccine rollout would be insufficient to prevent the exacerbation of cases, hospitalizations and deaths expected as the novel, more contagious SARS-CoV-2 variants become dominant. Bolstering the production and distribution of COVID-19 vaccines is critical to reducing severe health outcomes and preventing additional COVID-19 waves that could be fuelled by waning adherence to NPIs.

## Author contributions

Conceptualization: SMM, PJH, PS, APG, Data curation: TNV, SMM, Formal analysis: TNV, SMM, PS, Funding acquisition: SMM, MCF, APG, Investigation: All authors, Methodology: TNV, SMM, SP, Project administration: SMM, APG, PJH, Supervision: SMM, APG, Validation and Visualization: SMM, TNV, PS, Writing - original draft: All authors, Writing - review & editing: All authors

## Data sharing

All simulation codes are available at: https://github.com/thomasvilches/two_strains.

## Funding

SMM was supported by the Canadian Institutes of Health Research [OV4 – 170643, COVID-19 Rapid Research]. MCF was supported by the National Institutes of Health grant K01AI141576. APG and PS were funded by NSF Expeditions grant 1918784, NIH grant 1R01AI151176-01, National Science Foundation grant RAPID-2027755, Centers for Infectious Disease Control and Prevention grant U01IP001136 and the Notsew Orm Sands Foundation.

## Declaration of Competing Interest

All authors have nothing to disclose.

## Appendix Supplementary materials

Image, application 1
