# Supplementary material for: Accelerated vaccine rollout is imperative to mitigate highly transmissible COVID-19 variants
Source: eClinicalMedicine. 2021 Apr 25;35:100865. doi: 10.1016/j.eclinm.2021.100865 (PMC8072134; doi:10.1016/j.eclinm.2021.100865)
Supplement: Supplementary file 1 [file mmc1.docx]

**Appendix**

**Accelerated vaccine rollout is imperative to mitigate highly transmissible COVID-19 variants**

**Pratha Sah, PhD^1*^, Thomas Vilches, PhD^2*^, Seyed M. Moghadas^2*^, PhD^2^, Meagan C. Fitzpatrick, PhD^1,3^, Burton H. Singer^4^, PhD, Peter J. Hotez^5^, MD, PhD, Alison P. Galvani, PhD^1^**

^1^ Center for Infectious Disease Modeling and Analysis (CIDMA), Yale School of Public Health, New Haven, Connecticut, USA

^2^ Agent-Based Modelling Laboratory, York University, Toronto, Ontario, M3J 1P3 Canada

^3^ Center for Vaccine Development and Global Health, University of Maryland School of Medicine, 685 W Baltimore St, Baltimore, MD 21201, USA

^4^ Emerging Pathogens Institute, University of Florida, Gainesville, FL 32610, USA

^5^ National School of Tropical Medicine, Baylor College of Medicine, Houston, TX

This appendix provides further details of model parameterization, and additional results of the sensitivity analyses.

**Table A1.** Percentage of population in different age groups with and without comorbidities, including cardiovascular disease, diabetes, asthma, chronic obstructive pulmonary disease, hypertension, and cancer.

| **Condition** | **0-4** | **5-19** | **20-49** | **50-64** | **65+** | **All age groups** |
| --- | --- | --- | --- | --- | --- | --- |
| With comorbidity | 5% | 10% | 28% | 55% | 78% | 37% |
| Without comorbidity | 95% | 90% | 72% | 45% | 22% | 63% |
| **Fraction of total population** | 5.9% | 18.9% | 39.6% | 18.9% | 16.6% | 100% |

**Table A2.** Mixing patterns and the daily number of contacts derived from empirical observations. Daily numbers of contacts were sampled from negative binomial distributions for different scenarios.

| **Age group** | **Proportion of contacts between age groups** | | | | | **Daily number of contacts without interventions**  **Mean (SD)** | **Daily number of contacts with interventions of self-isolation**  **Mean (SD)** |
| --- | --- | --- | --- | --- | --- | --- | --- |
|  | **0-4** | **5-19** | **20-49** | **50-65** | **65+** |  |  |
| **0-4** | 0.2287 | 0.1839 | 0.4219 | 0.1116 | 0.0539 | 10.21 (7.65) | 2.86 (2.14) |
| **5-19** | 0.0276 | 0.5964 | 0.2878 | 0.0591 | 0.0291 | 16.793 (11.7201) | 4.70 (3.28) |
| **20-49** | 0.0376 | 0.1454 | 0.6253 | 0.1423 | 0.0494 | 13.795 (10.5045) | 3.86 (2.95) |
| **50-65** | 0.0242 | 0.1094 | 0.4867 | 0.2723 | 0.1074 | 11.2669 (9.5935) | 3.15 (2.66) |
| **65+** | 0.0207 | 0.1083 | 0.4071 | 0.2193 | 0.2446 | 8.0027 (6.9638) | 2.24 (1.95) |

**Table A3.** Estimated vaccine efficacies with associated timelines.

| **Vaccine efficacy** | **Week after the first dose** | | **Week after the second dose** | |
| --- | --- | --- | --- | --- |
| Pfizer-BioNTech | 1-2 | 3 | 1 | >1 |
| Infection | None | 46% (40% - 51%) | 60% (53% - 66%) | 92% (88% - 95%) |
| Symptomatic disease | None | 57% (50% - 63%) | 66% (57% - 73%) | 94% (87% - 98%) |
| Severe disease | None | 62% (39% - 80%) | 80% (59% - 94%) | 92% (75% - 100%) |
| Moderna | 1-2 | 3-4 | 1-2 | >2 |
| Infection | None | 61% (31% – 79%) | 61% (31% – 79%) | 93.5% (85.2% - 97.2%) |
| Symptomatic disease | None | 92.1% (68.8% - 99.1%) | 92.1% (68.8% - 99.1%) | 94.1% (89.3% - 96.8%) |
| Severe disease | None | 92.1% (68.8% - 99.1%) | 92.1% (68.8% - 99.1%) | 100% |

**Table A4.** Risk of death due to COVID-19.

|  | Age groups | | | | | | |
| --- | --- | --- | --- | --- | --- | --- | --- |
| **Hospitalized cases** | **0-19** | **20-44** | **45-54** | **55-64** | **65-74** | **75-84** | **85-100** |
| Non-ICU | 0.1% | 0.15% | 0.65% | 1.0% | 2.0% | 7.35% | 38.0% |
| ICU | 0.2% | 0.22% | 0.8% | 2.2% | 4.0% | 8.0% | 40.0% |

**Table A5.** Projected mean hospitalizations and deaths per 10000 population, with 95% credible intervals, for different scenarios of vaccine doses per day and transmissibility of a SGTF variant, over a time horizon of 300 days after the start of vaccination.

| **Vaccination rate per day** | **Total hospitalizations (95% CrI)** | | | | | |
| --- | --- | --- | --- | --- | --- | --- |
|  | RT=10% | RT=20% | RT=30% | RT=40% | RT=50% | RT=70% |
| 0 (no vaccination) | 14.6  (13.4, 15.8) | 26.9  (24.9, 29.1) | 45.9  (42.7, 48.9) | 65.7  (61.6, 69.6) | 88.9  (83.8, 93.9) | 131.9  (126.5, 138.2) |
| 1 million | 4.7  (4.4, 5.1) | 6.5  (6.0, 7.0) | 10.9  (10.0, 11.7) | 22.5  (21.0, 24.0) | 35.7  (33.4, 38.0) | 66.9  (63.4, 70.5) |
| 2 million | 4.2  (3.8, 4.5) | 5.2  (4.8, 5.6) | 7.7  (7.1, 8.2) | 14.8  (13.8, 15.8) | 24.0  (22.4, 25.6) | 49.5  (46.8, 52.2) |
| 3 million | 4.0  (3.6, 4.3) | 4.6  (4.3, 4.9) | 6.3  (5.8, 6.7) | 11.1  (10.4, 11.8) | 18.3  (17.1, 19.5) | 39.0  (36.7, 41.2) |
|  | **Total deaths (95% CrI)** | | | | | |
| 0 (no vaccination) | 4.1  (3.8, 4.5) | 9.0  (8.2, 9.7) | 16.7  (15.5, 17.9) | 25.6  (23.9, 27.3) | 35.9  (33.8, 37.9) | 57.2  (54.6, 60.0) |
| 1 million | 1.1  (1.0, 1.2) | 1.7  (1.5, 1.8) | 3.1  (2.8, 3.3) | 6.8  (6.2, 7.3) | 12.1  (11.3, 12.9) | 25.5  (24.1, 27.0) |
| 2 million | 0.9  (0.8, 1.0) | 1.2  (1.1, 1.3) | 2.0  (1.8, 2.2) | 4.1  (3.7, 4.4) | 7.1  (6.6, 7.7) | 16.7  (15.8, 17.8) |
| 3 million | 0.9  (0.8, 1.0) | 1.1  (1.0, 1.2) | 1.6  (1.5, 1.7) | 2.9  (2.7, 3.2) | 5.2  (4.8, 5.6) | 12.4  (11.7, 13.2) |


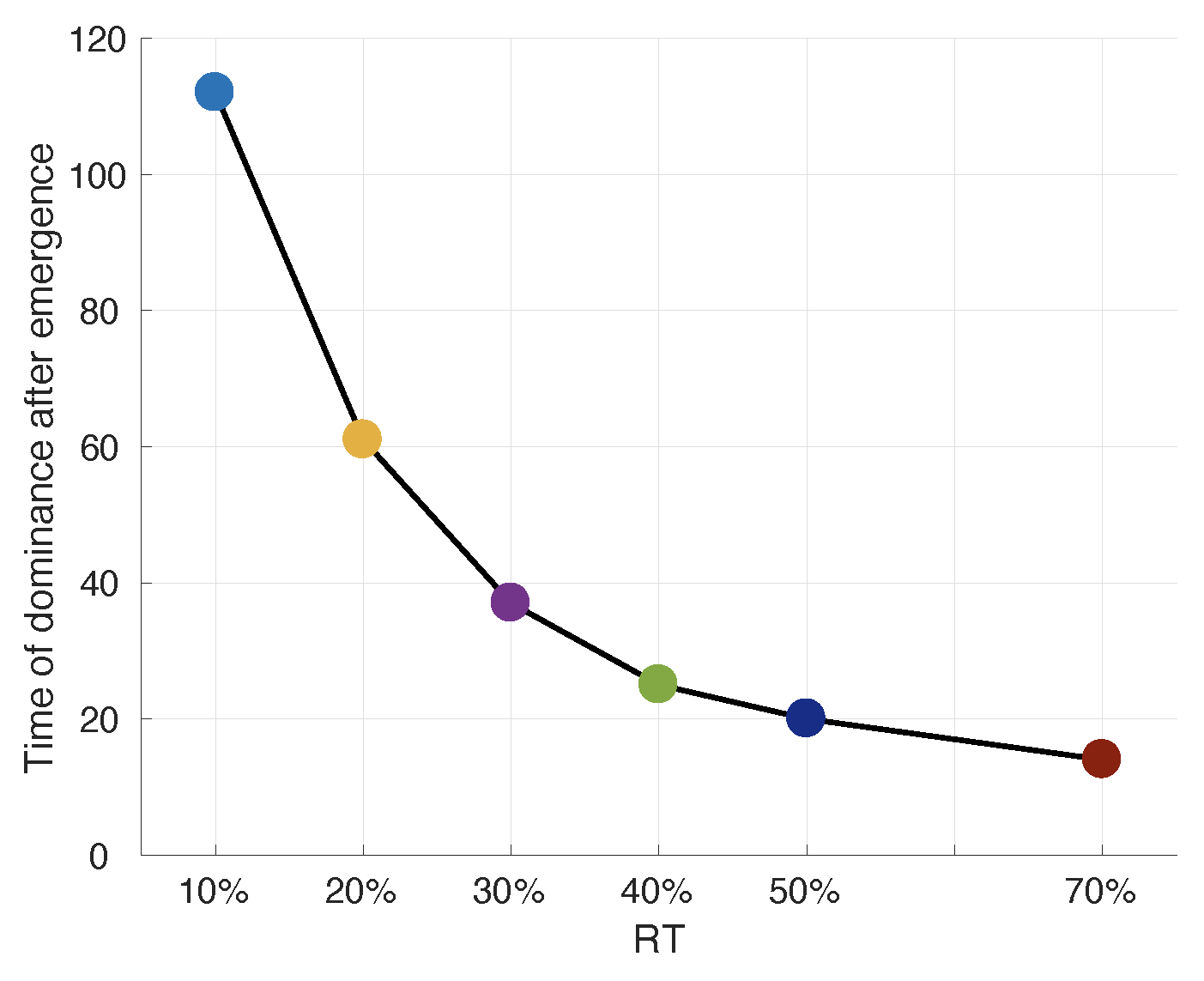


**Figure A1:** Time to dominance from the introduction of a variant with SGTF into the population.


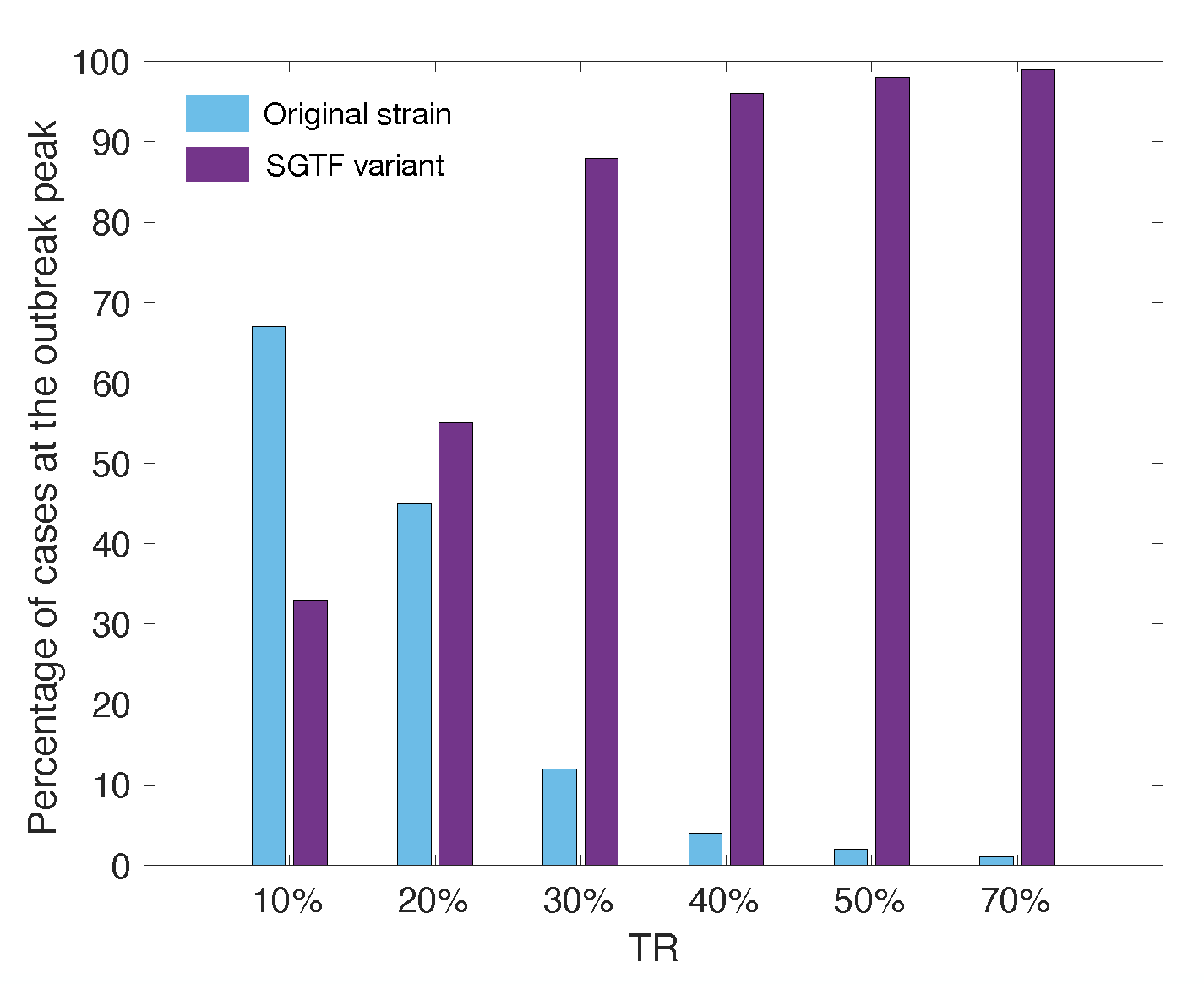


**Figure A2.** Percentage of cases at the peak of outbreak attributed to the original strain and SGTF variants with different RT.

**Results with Moderna vaccines and reduced efficacy against variants with SGTF**

**
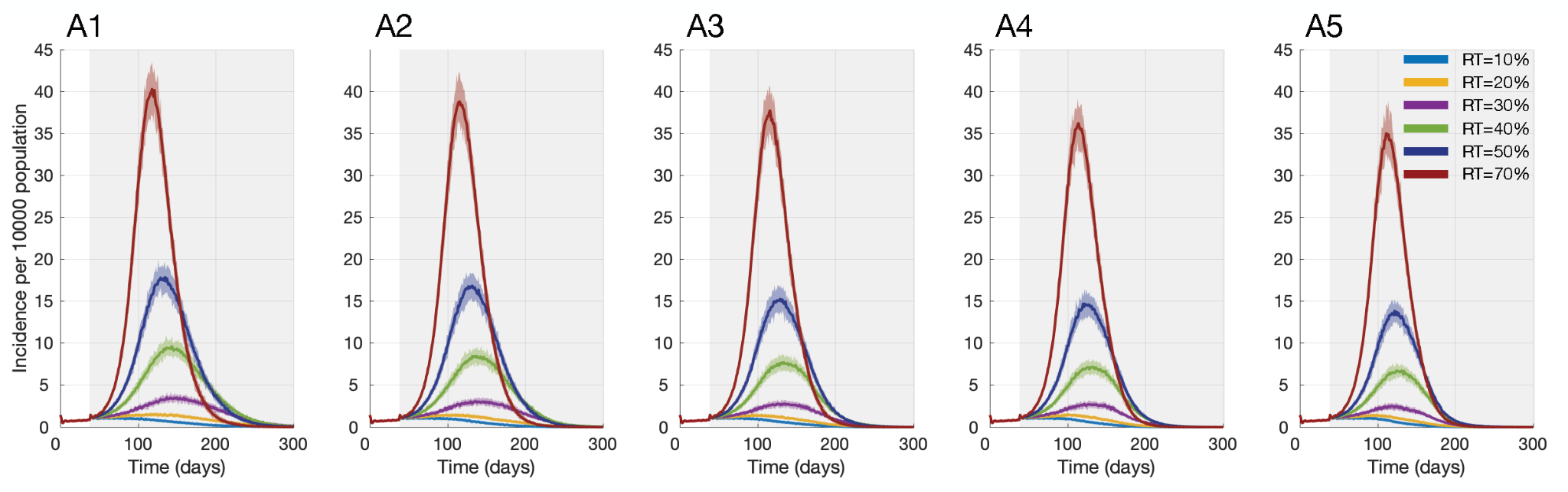
**

**Figure A3.** Projected incidence of infection per 10,000 population from the start of vaccination with different relative transmissibility of SGTF variants. Maximum daily Moderna vaccines administered are either (A1) 1 million; (A2) 1.5 million; (A3) 2 million; (A4) 2.5 million; or (A5) 3 million doses, per 10000 population. Vaccine efficacy against variants with SGTF was reduced by 20% relative to its estimated efficacy against the original strain.

**
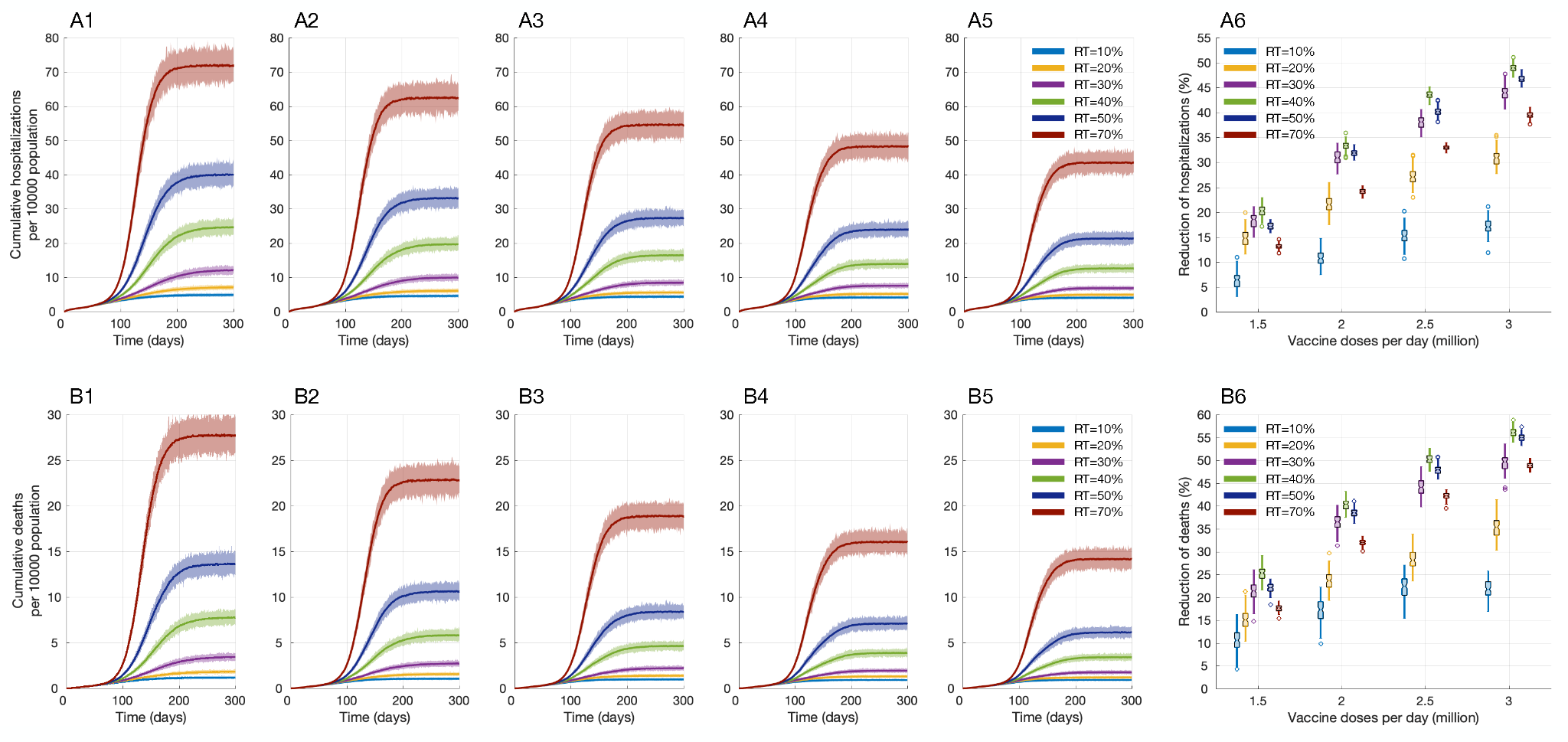
**

**Figure A4.** Projected cumulative hospitalizations (A1-A5) and deaths (B1-B5) per 10000 population for 300 days since the start of vaccination with different relative transmissibility of SGTF variants. Vaccine rollouts are with (A1,B1) 1 million; (A2,B2) 1.5 million; (A3,B3) 2 million; (A4,B4) 2.5 million; and (A5,B5) 3 million doses per day. Panels A6 and B6 represent the reduction of hospitalizations and deaths achieved by increasing the number of daily Moderna vaccine doses from 1 to 3 million doses. Vaccine efficacy against variants with SGTF was reduced by 20% relative to its estimated efficacy against the original strain.

**
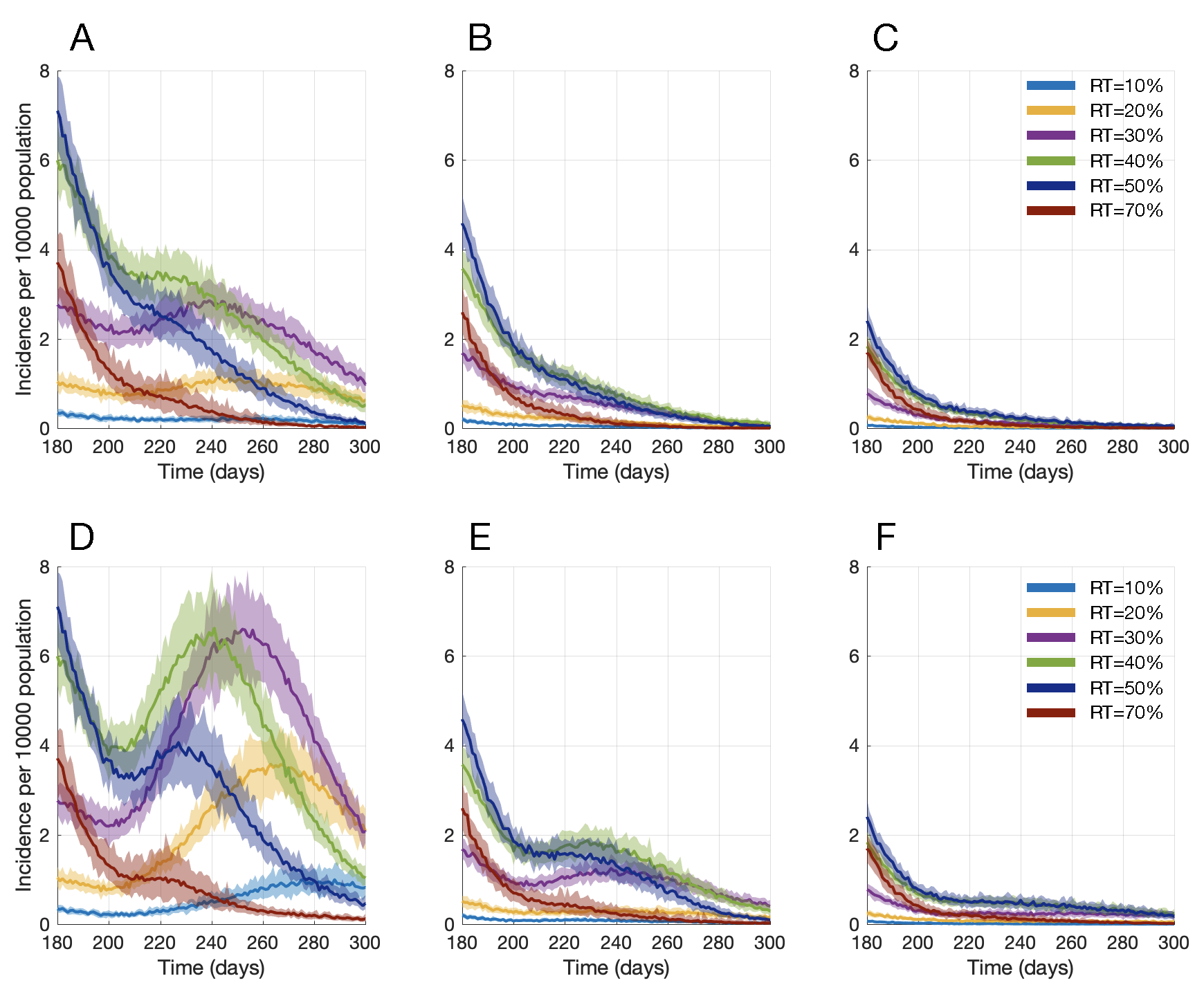
**

**Figure A5.** Projected incidence of infection per 10000 population with different relative transmissibility of SGTF variants. The number of daily contacts increased by 20% (A,B,C), and 40% (D,E,F) 200 days after the start of vaccination. Maximum daily Moderna vaccines administered are either 1 million (A,D); 2 million (B,E); or 3 million doses (C,F) in the entire US population. Vaccine efficacy against variants with SGTF was reduced by 20% relative to its estimated efficacy against the original strain.

**Results with Pfizer-BioNTech vaccines**


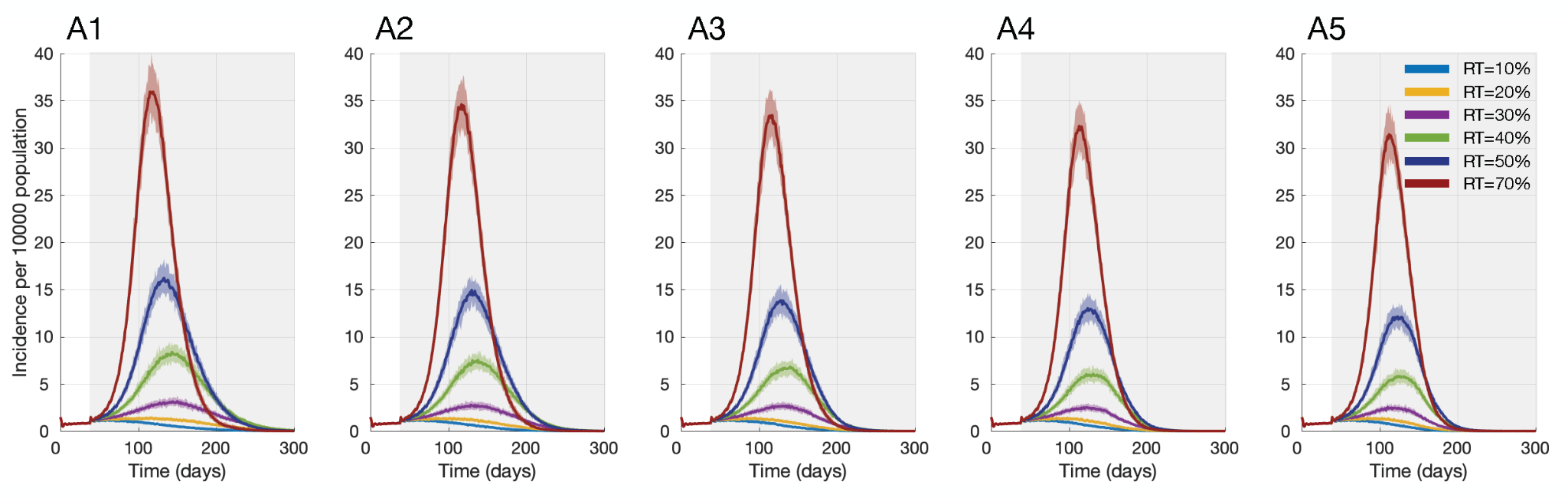


**Figure A6.** Projected incidence of infection per 10000 population from the start of vaccination with different relative transmissibility of SGTF variants. Maximum daily Pfizer-BioNTech vaccines administered are either (A1) 1 million; (A2) 1.5 million; (A3) 2 million; (A4) 2.5 million; or (A5) 3 million doses. Vaccine efficacy was assumed to be the same against the original strain and variants with SGTF


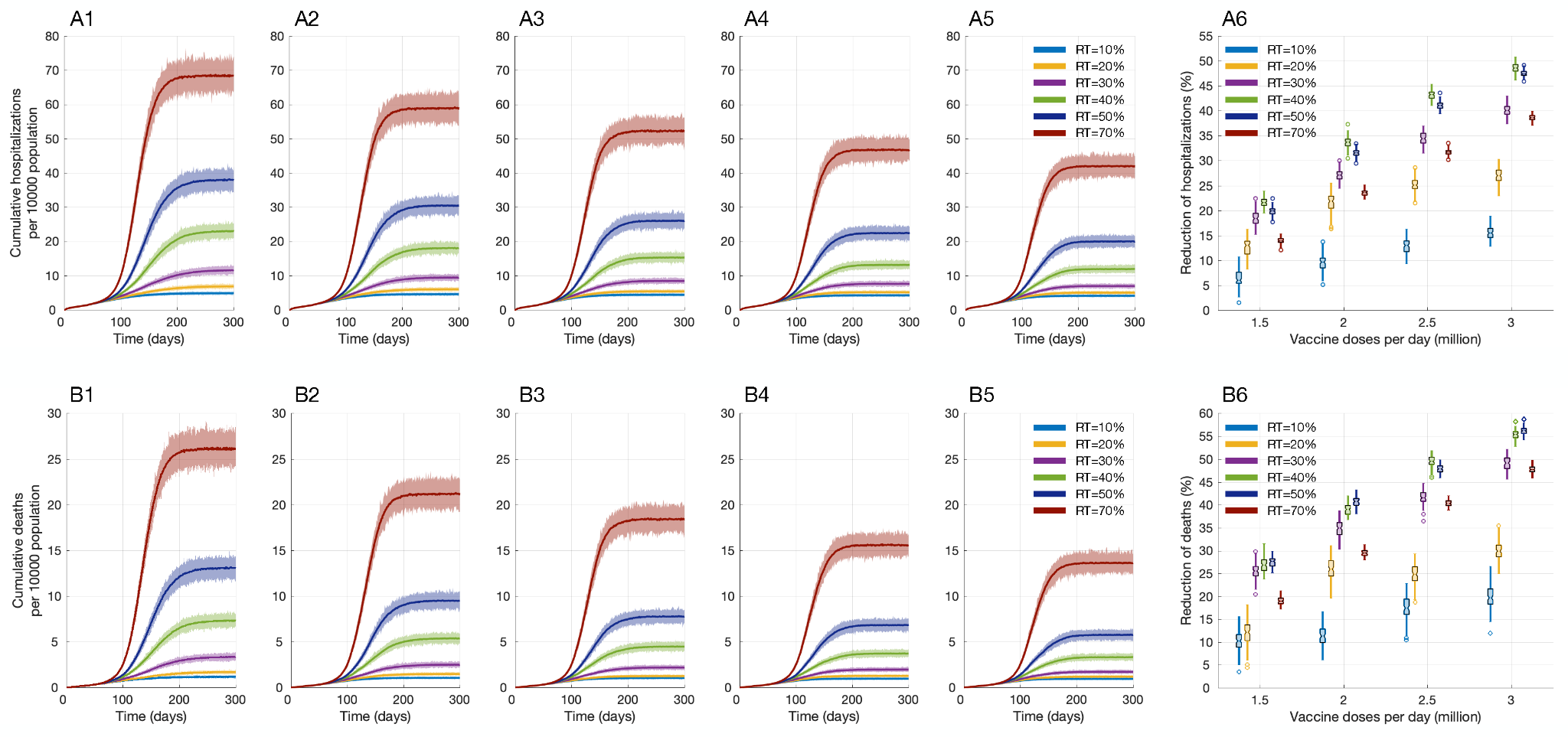


**Figure A7.** Projected cumulative hospitalizations (A1-A5) and deaths (B1-B5) per 10000 population since the start of vaccination with different relative transmissibility of SGTF variants. Vaccine rollouts are with (A1,B1) 1 million; (A2,B2) 1.5 million; (A3,B3) 2 million; (A4,B4) 2.5 million; and (A5,B5) 3 million doses per day in the entire US population. Panels A6 and B6 represent the reduction of hospitalizations and deaths achieved by increasing the number of daily Pfizer-BioNTech vaccine doses from 1 to 3 million doses. Vaccine efficacy was assumed to be the same against the original strain and variants with SGTF


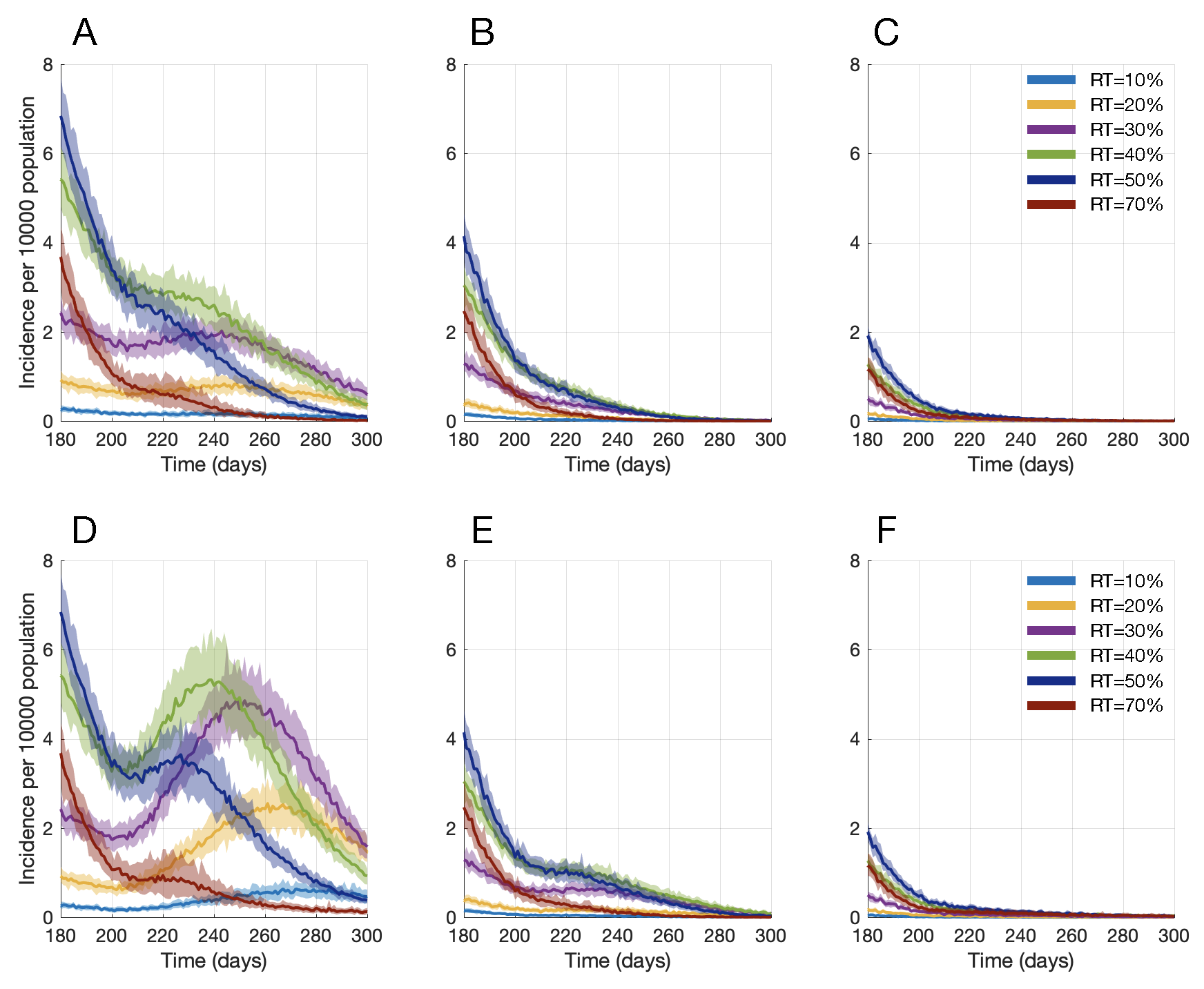


**Figure A8.** Projected incidence of infection per 10000 population with different relative transmissibility of SGTF variants. The number of daily contacts increased by 20% (A,B,C), and 40% (D,E,F) 200 days after the start of vaccination. Maximum daily Pfizer-BioNTech vaccines administered are either 1 million (A,D); 2 million (B,E); or 3 million doses (C,F) in the entire US population. Vaccine efficacy was assumed to be the same against the original strain and variants with SGTF.

**Results with Pfizer-BioNTech vaccines** **and reduced efficacy against variants with SGTF**


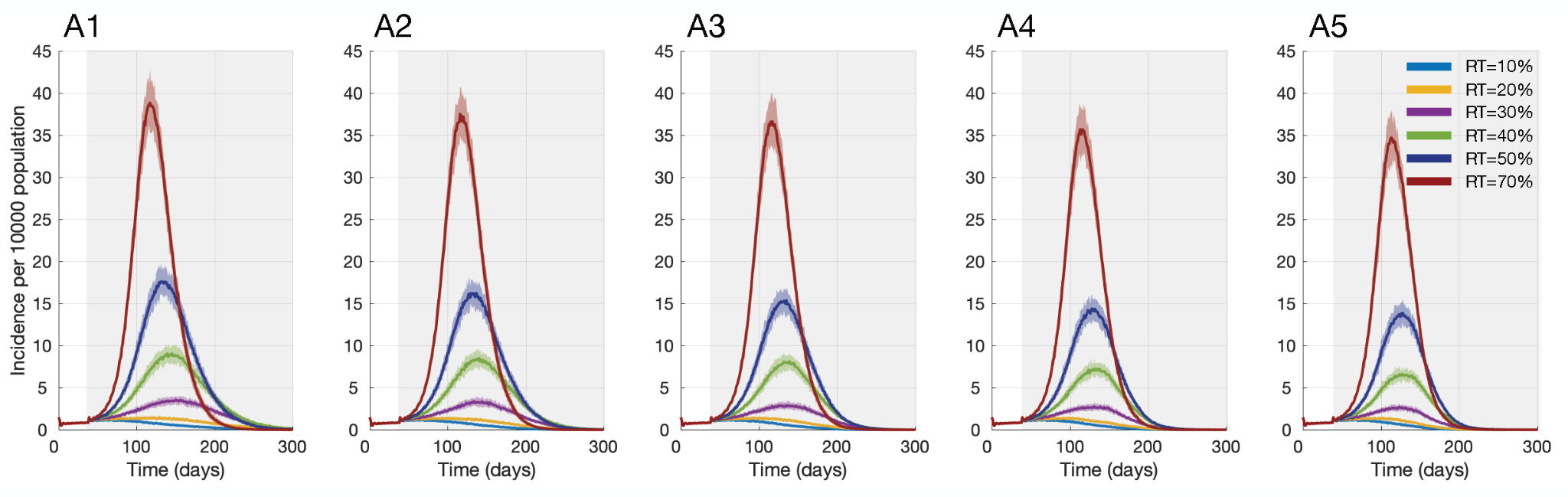


**Figure A9.** Projected incidence of infection per 10000 population from the start of vaccination with different relative transmissibility of SGTF variants. Maximum daily Pfizer-BioNTech vaccines administered are either (A1) 1 million; (A2) 1.5 million; (A3) 2 million; (A4) 2.5 million; or (A5) 3 million doses. Vaccine efficacy against variants with SGTF was reduced by 20% relative to its estimated efficacy against the original strain.


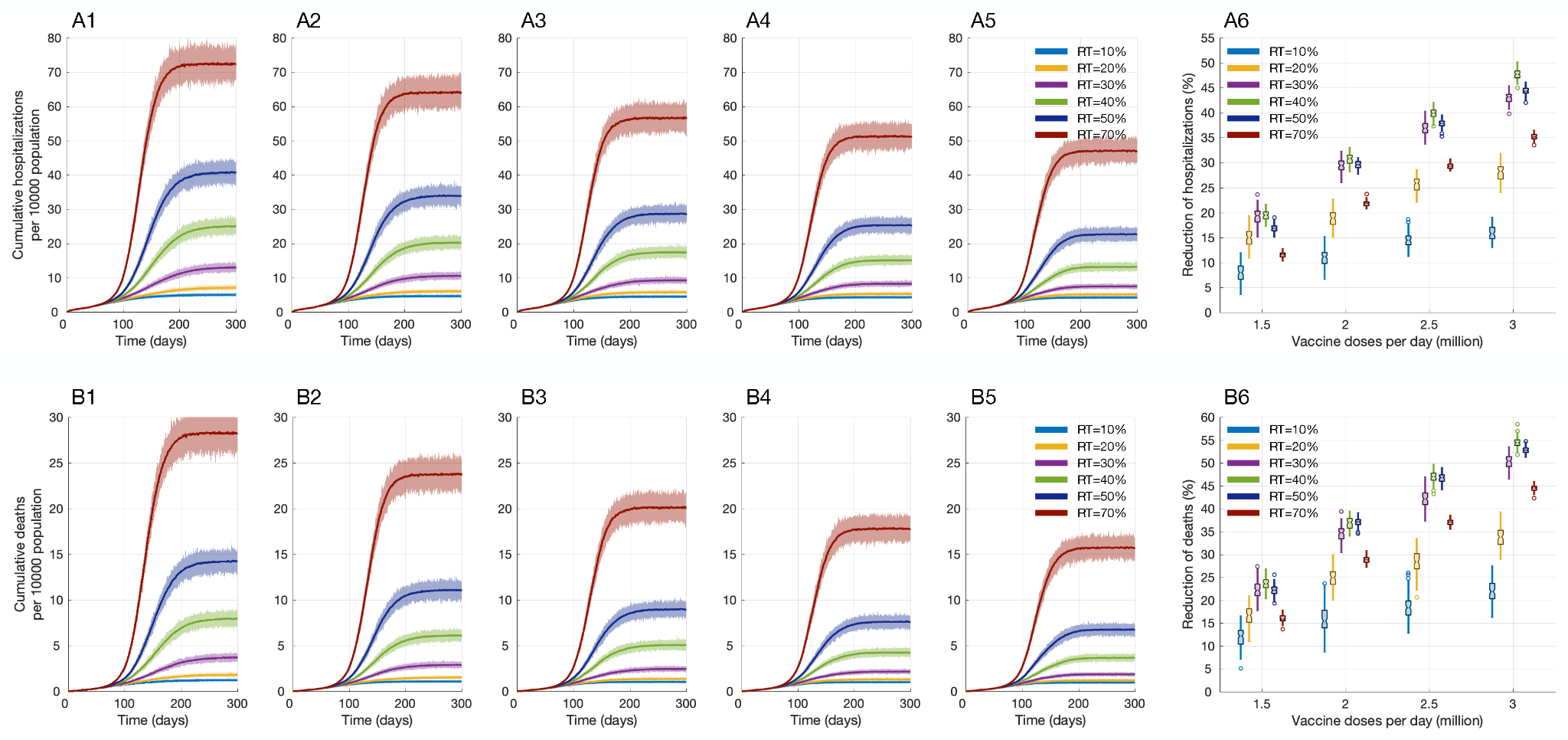


**Figure A10.** Projected cumulative hospitalizations (A1-A5) and deaths (B1-B5) per 10000 population since the start of vaccination with different relative transmissibility of SGTF variants. Vaccine rollouts are with (A1,B1) 1 million; (A2,B2) 1.5 million; (A3,B3) 2 million; (A4,B4) 2.5 million; and (A5,B5) 3 million doses per day in the entire US population. Panels A6 and B6 represent the reduction of hospitalizations and deaths achieved by increasing the number of daily Pfizer-BioNTech vaccine doses from 1 to 3 million doses. Vaccine efficacy against variants with SGTF was reduced by 20% relative to its estimated efficacy against the original strain.


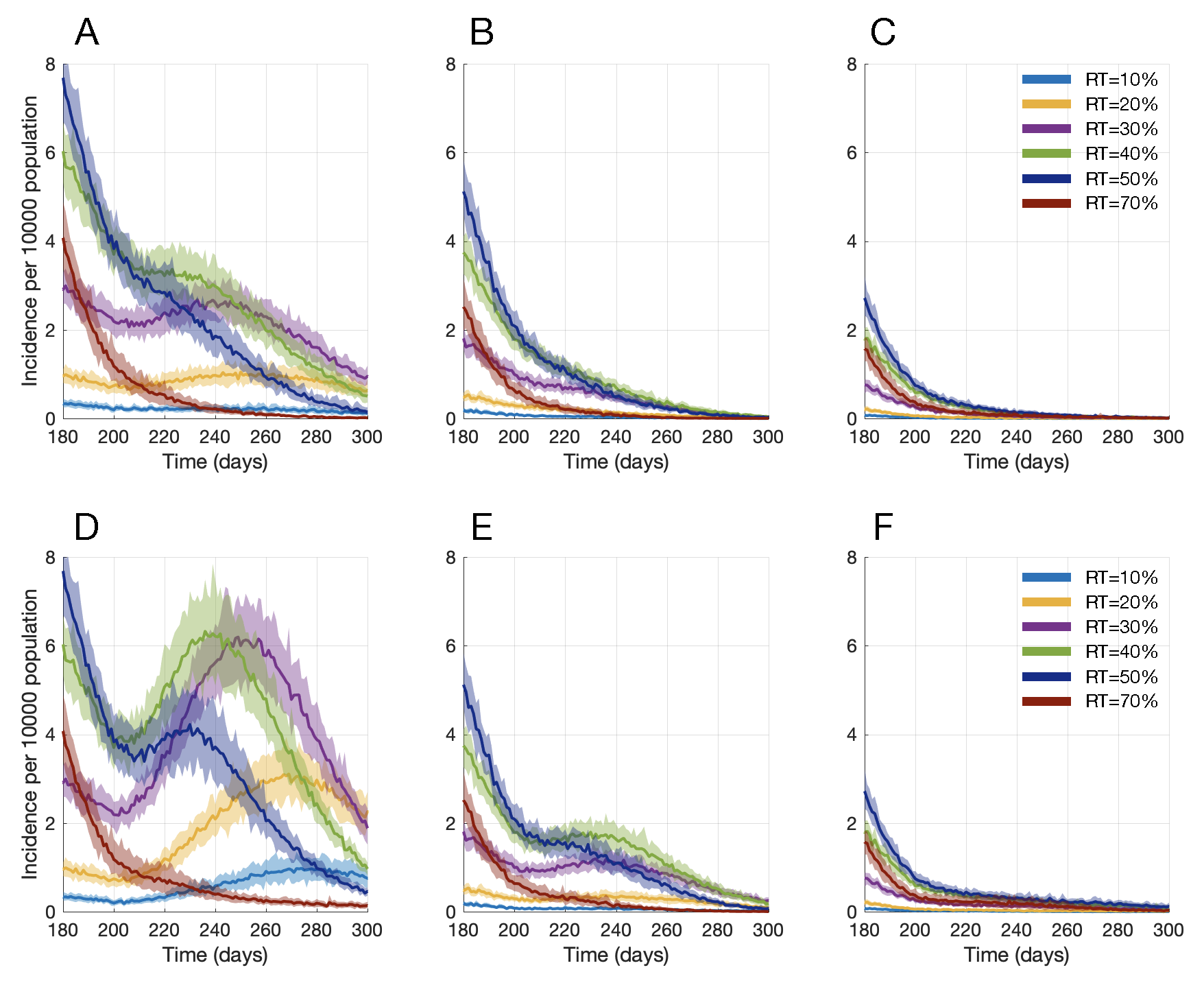


**Figure A10.** Projected incidence of infection per 10000 population with different relative transmissibility of SGTF variants. The number of daily contacts increased by 20% (A,B,C), and 40% (D,E,F) 200 days after the start of vaccination. Maximum daily Pfizer-BioNTech vaccines administered are either 1 million (A,D); 2 million (B,E); or 3 million doses (C,F) in the entire US population. Vaccine efficacy against variants with SGTF was reduced by 20% relative to its estimated efficacy against the original strain.
